# Design of Decision Tree Structure with Improved BPNN Nodes for High-Accuracy Locomotion Mode Recognition Using a Single IMU

**DOI:** 10.3390/s21020526

**Published:** 2021-01-13

**Authors:** Yang Han, Chunbao Liu, Lingyun Yan, Lei Ren

**Affiliations:** 1 The School of Mechanical Science and Aerospace Engineering, Jilin University, Changchun 130000, China; hanyang19@mails.jlu.edu.cn; 2 Key Laboratory of Bionic Engineering, Ministry of Education, Jilin University, Changchun 130000, China; 3 The School of Mechanical, Aerospace and Civil Engineering, University of Manchester, Manchester M13 9PL, UK; lingyun.yan@manchester.ac.uk

**Keywords:** wearable robotic system, locomotion mode recognition, inertial measurement unit (IMU), decision tree structure (DTS)

## Abstract

Smart wearable robotic system, such as exoskeleton assist device and powered lower limb prostheses can rapidly and accurately realize man–machine interaction through locomotion mode recognition system. However, previous locomotion mode recognition studies usually adopted more sensors for higher accuracy and effective intelligent algorithms to recognize multiple locomotion modes simultaneously. To reduce the burden of sensors on users and recognize more locomotion modes, we design a novel decision tree structure (DTS) based on using an improved backpropagation neural network (IBPNN) as judgment nodes named IBPNN-DTS, after analyzing the experimental locomotion mode data using the original values with a 200-ms time window for a single inertial measurement unit to hierarchically identify nine common locomotion modes (level walking at three kinds of speeds, ramp ascent/descent, stair ascent/descent, Sit, and Stand). In addition, we reduce the number of parameters in the IBPNN for structure optimization and adopted the artificial bee colony (ABC) algorithm to perform global search for initial weight and threshold value to eliminate system uncertainty because randomly generated initial values tend to result in a failure to converge or falling into local optima. Experimental results demonstrate that recognition accuracy of the IBPNN-DTS with ABC optimization (ABC-IBPNN-DTS) was up to 96.71% (97.29% for the IBPNN-DTS). Compared to IBPNN-DTS without optimization, the number of parameters in ABC-IBPNN-DTS shrank by 66% with only a 0.58% reduction in accuracy while the classification model kept high robustness.

## 1. Introduction

In recent years, with the rapid development of wearable robotic system, many new intelligent products have been introduced, e.g., exoskeleton assist device and powered lower limb prosthesis. These products can effectively improve the athletic ability of healthy people and recover the daily life ability of the disabled.

The intelligence of wearable robotic system is reflected in the fact that such devices, e.g., Rewalk Personal 6.0 from Rewalk Robotics and Genium X3 from Ottobock can understand human thoughts and realize corresponding functions according to human intent. Therefore, the development of locomotion mode recognition determines the upper limit of wearable robotic system, which is the most critical link relative to elevating a machine into a robot.

Obtaining valid data from sensors is the first step in human locomotion mode recognition. Current studies generally consider electromyographic (EMG) signals [1,2,3,4,5], which can reflect neuromuscular activity to a certain extent, and mechanical signals [6,7,8,9], which can be collected using various sensors, e.g., inertial measurement unit (IMU), located throughout the body and load cells placed at the bottom of the foot. Several studies have combined EMG and mechanical signals to realize locomotion mode recognition [10,11,12]. In addition, environmental information has been proven effective relative to improving the accuracy of locomotion mode recognition [13,14,15].

After obtaining reliable data, the performance of algorithms is always key to locomotion mode recognition. For three common human locomotion modes, i.e., level walking, ramp ascent, and ramp descent, Uriel et al. used wearable sensors on healthy humans to identify locomotion modes based on a probabilistic Bayesian approach with a sequential analysis method [16]. Feng et al. developed the strain gauge potential in locomotion mode recognition, and the average accuracy reached 92.53% using a convolutional neural network to evaluate the movement state with the original data as input [17]; however, three locomotion modes are not representative of the total range of human locomotion. Thus, two additional locomotion modes, i.e., stair ascent and descent, were added to the research. Liu et al. [1] employed a kernel principal component analysis algorithm to perform dimensionality reduction on the EMG eigenvalues of five lower limb muscles in a healthy human body. Then, they designed a classification model based on a relevance vector machine for identification, and the accuracy of this method reached 96.67%. Zhao et al. placed an IMU and two load cells in the receiving cavity of an active lower limb prosthesis to collect locomotion information, and they adopted a hidden Markov model (HMM) [18] for analysis and identification. The experimental results demonstrated a total recognition rate of 96%, which provided a basis for the development of an active prosthesis.

Based on steady-state motion, some studies have focused on transitional motion. Wang, Liu, and Sheng used a support vector machine (SVM) [19], long short-term memory [20], and a Gaussian mixture model-hidden Markov model [21], respectively to recognize locomotion modes using three IMUs positioned on the lower limbs, and all three algorithms demonstrated high recognition rates. Zheng et al. [22] used quadratic discriminant analysis (QDA) and an SVM as recognition models to distinguish 10 transitional motions using data from an IMU and a load cell on six transtibial amputees’ ankle prostheses. The experimental results demonstrated that the accuracy of both algorithms was greater than 94%. Ali et al. adopted a unique ear-mounted sensor for data collection. They applied a recursive map classification algorithm to identify different transitional motions. Their experimental results proved that a framework for the categorization and analysis of transitions in manifold space [23] has good application in damage and post-surgery recovery research. Parri et al. [24] applied a time-based approach based on gait kinematics data to classify steady-state and transitional motion, and they designed event-based fuzzy-logic rules triggered by a minimal set of relevant biomechanical features of a load cell to classify transitional motions. The recognition results demonstrated that this algorithm provides an effective reference value for locomotion mode recognition of lower limb wearable robots. By analyzing experiments performed by transtibial amputees, Su [9] and Young [25] found that user-dependent classification was more accurate than user-independent classification, proving that locomotion mode recognition algorithms are more effective in terms of individual training.

For using a single IMU, in [26], translational signals which were derived from the inertial signals including accelerations, velocities, and displacements of a transtibial prosthesis, can enhance the walking task identification with an error reduction of 6.8% in five locomotion modes. Bartlett and Goldfarb [27] proposed to identify the gait activities based on a feature set extracted from a phase-variable-based coordinate system. This method was more effective than linear discriminant analysis (LDA) when using nonsubject-specific training data of three locomotion modes from an IMU wore on the thigh. Gao [28] proposed a terrain geometry-based algorithm with an elliptical boundary as trigger condition using the inclination grade of the ground to classify five locomotion modes with an IMU estimating the foot trajectory.

In summary, previous studies have generally used multiple sensors to collect EMG and mechanical signals for one-time recognition of locomotion modes. Increasing the number of sensors can improve locomotion mode recognition accuracy [18,22]. However, using too many sensors on wearable robotic system, especially power lower limb prosthesis, will impose extra burden on the user’s body and affect the effectiveness of movement. In addition, when using a small amount of sensors like an IMU, the number of locomotion modes increases and similar locomotion modes occur, e.g., level walking at different speeds [27] and ramp motions at small angles [28] may lead to significant errors and reduced model accuracy.

In this study, we placed a single IMU under the knee joint to monitor nine locomotion modes of the participants. In addition, to identify the nine locomotion modes, we designed a new multi-layer decision tree structure (DTS) based on experimental data, and we improved the backpropagation neural network (IBPNN), which has obtained certain results in the artificial intelligence field [29,30]. The IBPNN algorithm was used as the judgment node of the DTS to constitute an IBPNN-DTS classification model. Finally, the ABC-IBPNN-DTS classification model, which has similar accuracy but fewer number of parameters than the IBPNN-DTS, was constructed after optimizing the number of neurons in the hidden layer of the IBPNN and selecting the optimal initial weight and threshold value using the ABC algorithm. The experimental results demonstrated that the ABC-IBPNN-DTS model has high-accurate identification ability. As a result, the ABC-IBPNN-DTS classification model based on a single IMU is of great guiding significance for the intelligentization of wearable robotic system, especially active lower limb prosthesis.

## 2. Proposed Methods

### 2.1. Definition of Locomotion Modes

When users employ smart wearable robotic system, e.g., exoskeleton boosters and powered lower limb prostheses, they must convey their ideas to the wearable device, and the effectiveness of the device depends on whether it can realize corresponding movements according to the user’s intent. Therefore, classification models based on intelligent algorithms can provide signals for wearable robotic system to identify nine common locomotion modes to realize rapid and accurate man–machine interaction.

Human movement is periodic and regular; therefore, it is helpful to select the nine typical human locomotion modes for pattern recognition to distinguish the current locomotion modes. In this study, the nine locomotion modes were defined as slow level walking (SLW), medium-speed level walking (MLW), fast level walking (FLW), ramp descent (RD), stair descent (SD), sit, stand, ramp ascent (RA), and stair ascent (SA). The velocity and experimental platform of each locomotion mode are shown in Table 1.

### 2.2. Locomotion Mode Data-Based DTS Design

A decision tree (DT) is a basic regression and classification method. In classification problems, the DT represents the process of classifying samples according to their characteristics and can be considered a set of if-then. A DT has a clear structure and low computational complexity; however, DT results are prone to overfitting. Representative examples of DT algorithms [31] include ID3, C4.5, CART, and the stochastic forest algorithm [32] derived from the DT, which are widely used in pattern recognition tasks [33].

**Figure 1:**
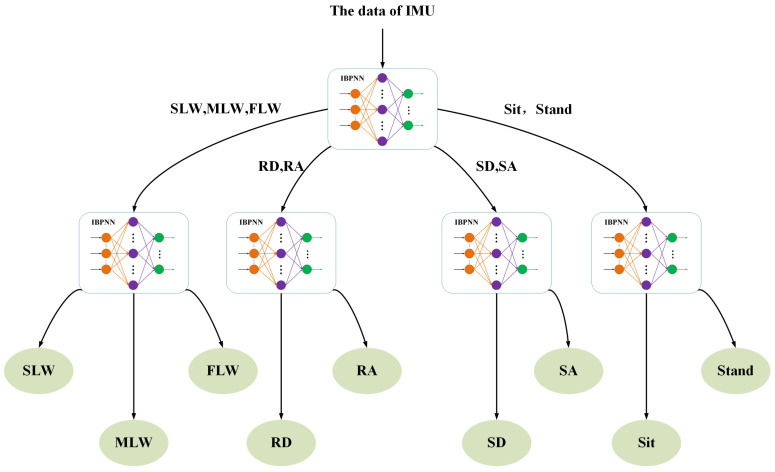
Decision tree structure (DTS) A: two layers and five judgment nodes based on locomotion mode data.

**Figure 2:**
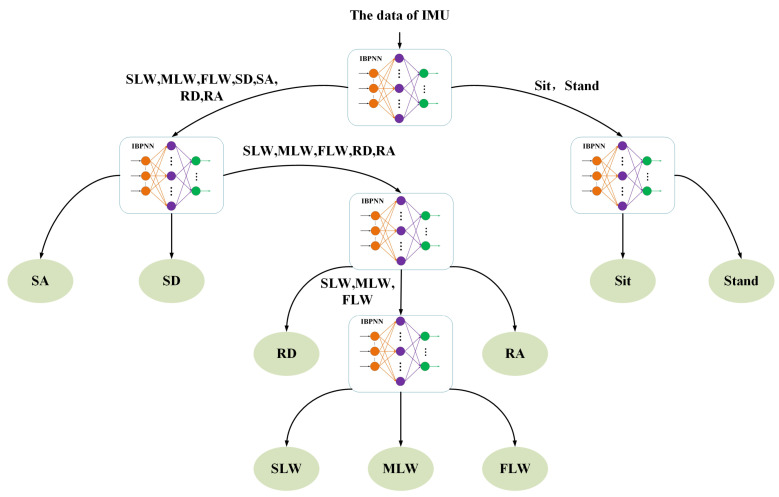
DTS B: four layers and five judgment nodes based on locomotion mode data.

Through observing the collection of human movement data and preliminary experiments, we found that the data within each categorie (a: SLW, MLW, FLW; b: RD, RA; c: SD, SA; d: sit, stand) has strong similarity and the data among four categories have certain differences. Given that, we have designed two DTSs to distinguish the nine locomotion modes hierarchically under the constraint of the minimum number of nodes, as shown in Figure 1 and Figure 2. The input to the DTS was IMU data, and the IBPNN algorithm was employed to determine the node evaluation criterion. In addition, the ABC algorithm was employed to optimize the initial weight and threshold value of the IBPNN algorithm.

### 2.3. IBPNN for DTS Judgment Node

The BPNN algorithm is one of the most important artificial neural network algorithms due to its unique mechanism of neural connections and its theoretical ability to fit any nonlinear function with a sufficient number of neurons. The BPNN algorithm has been widely used in pattern recognition and system modeling [34,35]. Compared to other algorithms, e.g., SVM, QDA, and LDA, the BPNN algorithm has a better effect in locomotion mode recognition [36]. In this study, the BPNN algorithm was improved by adaptive learning rate and forgetting factor which can accelerate the convergence, and the IBPNN algorithm as the DTS node has good identification ability and convergence speed.

The IBPNN consists of an input layer, a hidden layer, and an output layer, which are expressed as follows.

(1) $$x=\{x_{1},x_{2}\cdot \cdot \cdot x_{i}\}\;i=1,2\cdot \cdot \cdot n$$

(2) $$h_{j}=f\left(\sum_{i=1}^{n}w_{ij}*x_{i}-\theta_{j}\right)\;j=1,2\cdot \cdot \cdot m$$

(3) $$y_{k}=f\left(\sum_{j=1}^{m}w_{jk}*h_{j}-\theta_{k}\right)\;k=1,2\cdot \cdot \cdot l$$

Here, *n*, *m*, *l* represent the number of neurons in the input layer, the hidden layer, and the output layer. $x_{i}$ is the input to the IBPNN. $w_{ij}$ and $\theta_{j}$ are the weight and threshold value of the hidden layer, and $w_{jk}$ and $\theta_{k}$ are the weight and threshold value of the output layer, respectively. In addition, the sigmoid function, $f\left(x\right)=1/1+e^{-x}$, is employed as the activation function for both the hidden and output layers.

We consider the error function for the iteration of the weight and threshold value as follows:

(4) $$E_{k}=\frac{1}{2}\sum_{k=0}^{l}{\left(y_{k}-{\hat{y}}_{k}\right)}^{2},$$

where $y_{k}$ and ${\hat{y}}_{k}$ are the actual and theoretical outputs of the IBPNN, respectively.

The iteration of the weight and threshold value in the BPNN employs the traditional backpropagation algorithm with a low convergence rate. Thus, to accelerate the convergence speed of the BPNN, we improved the traditional backpropagation algorithm with adaptive learning rate α(t), and forgetting factor β which is a constant, where α(t) is expressed as follows.

(5) $$\left\{\begin{array}{c} \alpha \left(t\right)=0.99*\alpha \left(t-1\right)\;E_{k}\left(t\right)>1.04*E_{k}\left(t-1\right)\\ \alpha \left(t\right)=1.01*\alpha \left(t-1\right)\;E_{k}\left(t\right)<E_{k}\left(t-1\right)\\ \alpha (t)=\alpha (t-1)\;\;\;\;\;\;else \end{array}\right.$$

The value of forgetting factor β was obtained by trial and error and parameters of adaptive learning rate α(t) were based on the number of iterations. With α(t) and β, the iteration formulas of the weight and threshold value in the output and hidden layers are expressed as follows:

(6) $$\left\{\begin{array}{cc} \Delta w_{jk}\left(t\right) & =\alpha \left(t\right)*\left[h_{j}*\left({\hat{y}}_{k}-y_{k}\right)*y_{k}*\left(1-y_{k}\right)\right]\left(t\right)+\left[h_{j}*\left({\hat{y}}_{k}-y_{k}\right)*y_{k}*\left(1-y_{k}\right)\right]\left(t-1\right)\\ \; & *\beta *\alpha (t)\\ \Delta \theta_{k}\left(t\right) & =\alpha \left(t\right)*\left[\left({\hat{y}}_{k}-y_{k}\right)*y_{k}*\left(1-y_{k}\right)\right]\left(t\right)*\left(-1\right)+\left[\left({\hat{y}}_{k}-y_{k}\right)*y_{k}*\left(1-y_{k}\right)\right]\left(t-1\right)\\ \; & *(-1)*\beta *\alpha (t) \end{array}\right.$$

(7) $$\left\{\begin{array}{cc} \Delta w_{ij}\left(t\right) & =\alpha \left(t\right)*x_{i}\left(t\right)*\left[h_{j}*\left(1-h_{j}\right)*w_{jk}*\left({\hat{y}}_{k}-y_{k}\right)*y_{k}*\left(1-y_{k}\right)\right]\left(t\right)+\beta *\alpha \left(t\right)\\ \; & *x_{i}\left(t\right)*\left[h_{j}*\left(1-h_{j}\right)*w_{jk}*\left({\hat{y}}_{k}-y_{k}\right)*y_{k}*\left(1-y_{k}\right)\right]\left(t-1\right)\\ \Delta \theta_{j}\left(t\right) & =\alpha \left(t\right)*\left(-1\right)*\left[h_{j}*\left(1-h_{j}\right)*w_{jk}*\left({\hat{y}}_{k}-y_{k}\right)*y_{k}*\left(1-y_{k}\right)\right]\left(t\right)+\beta *\alpha \left(t\right)\\ \; & *\left(-1\right)*\left[h_{j}*\left(1-h_{j}\right)*w_{jk}*\left({\hat{y}}_{k}-y_{k}\right)*y_{k}*\left(1-y_{k}\right)\right]\left(t-1\right) \end{array}\right.$$

### 2.4. ABC for Optimizing Initial Parameters

ABC is a swarm intelligence optimization algorithm that simulates the foraging behavior of bees [37,38,39]. The ABC algorithm is frequently used for system identification and parameter optimization. Compared to other swarm intelligence algorithms, e.g., genetic algorithms, the ant colony algorithm [40], and particle swarm optimization algorithms [41], the most significant advantage of the ABC algorithm is that it combines global and local search in each iteration, which can help avoid falling into local optima. In this study, the ABC algorithm was employed to select the optimal initial weight and threshold value of the IBPNN.

The ABC algorithm comprises collecting, following, and scout bees. Collecting bees, which have the same location as nectar sources, search in the neighborhood for the optimal location of all nectar sources. Note that the degree of nectar sources depends on the value of a fitness function, and then the information of the nectar sources is transmitted to other bees. Following bees select correct nectar sources according to the value of a fitness function of nectar sources sent by the collecting bees. The main role of scout bees is to randomly search for a new location. The ABC algorithm can be realized in five steps.

1. Initialize the bee colony. For a *D*-dimension vector to be optimized, *N* feasible solutions are generated randomly, where *N* is the number of collecting and following bees. Feasible solutions $X_{i}^{j}$ are expressed as follows:
  (8) $$X_{i}^{j}=X_{\min}^{j}+rand\left(X_{\max}^{j}-X_{\min}^{j}\right)\;j=1,2,3\cdot \cdot \cdot D,$$
  where $X_{\max}^{j}$ and $X_{\min}^{j}$ are the maximum and maximum of the *D*-dimension vector, respectively.
2. Calculate fitness function fit. The fitness function fit is used to evaluate the quality of nectar sources based on the error function of the IBPNN.
  (9) $$fit=\min E_{k}=\frac{1}{2}\sum_{k=0}^{l}{\left(y_{k}-{\hat{y}}_{k}\right)}^{2}\;k=1,2,3\cdot \cdot \cdot l$$
3. Conduct local search at optimal nectar source locations. The search function is expressed as follows.
  (10) $$newX_{i}^{j}=X_{i}^{j}+rand\left(X_{i}^{j}-X_{k}^{j}\right)\;k=1,2,3\cdot \cdot \cdot N$$
  The bees replace a nectar source with a new location according to a greed criterion to ensure that the entire evolution process does not recede. The greedy selection operator is expressed as follows.
  (11) $$P\{newX_{i}\}=\left\{\begin{array}{c} 1\;f\left(newX_{i}\right)>=f\left(X_{i}\right)\\ 0\;f\left(newX_{i}\right)<f\left(X_{i}\right) \end{array}\right.$$
4. A following bee chooses to follow a collecting bee according to the nectar source information it receives based on a certain probability proportional to fitness.
  (12) $$P_{i}=\frac{fit_{i}}{\sum_{n=1}^{N}fit_{n}}$$
5. If a better nectar source is not found after collecting bees have finished the finite limit iterations (i.e., the local search), the first step is repeated until the total number of iterations (i.e., the global search) is completed to obtain the optimal *D*-dimension vector.

## 3. Experiments

### 3.1. Experimental Protocol

**Figure 3:**
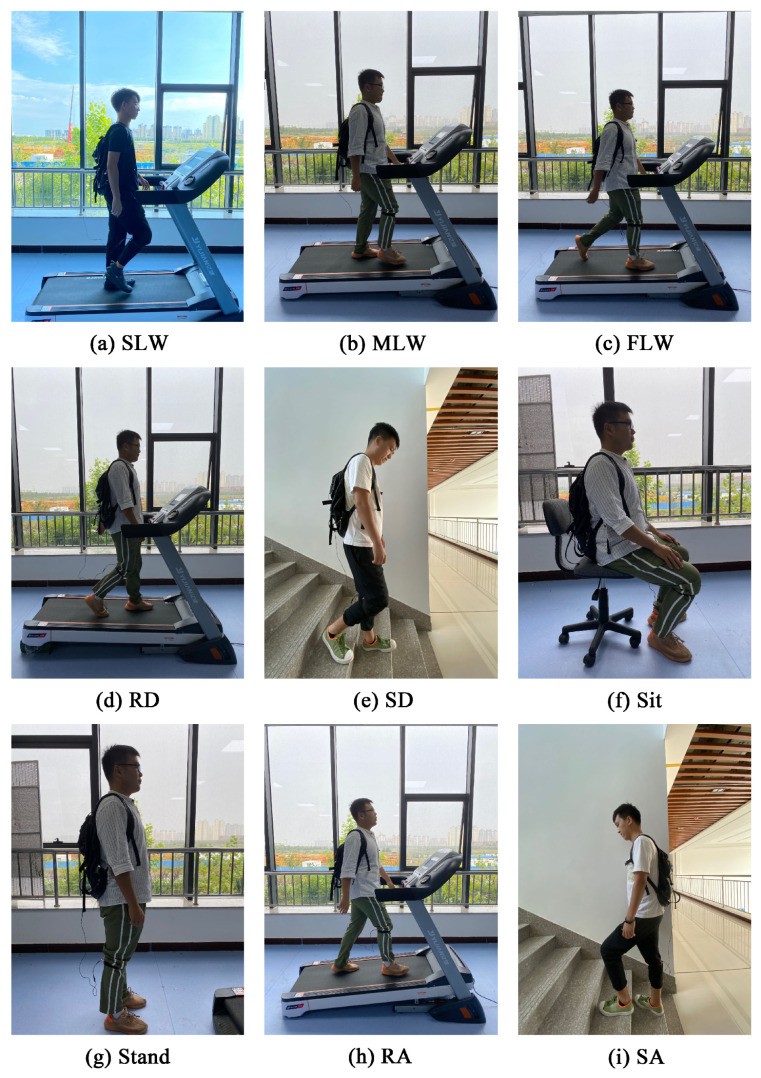
Data collection for locomotion modes.

Six healthy subjects varied in age (24–30 years), height (1.63–1.83 m), and weight (40–80 kg) participated in this experiment. Here, a single IMU was placed below the knee joint of the experimental subject, which is a common sensor position in active lower limb prostheses [42]. To facilitate data collection, the wireless socket communication of a Raspberry Pi was used to transmit data to the visualization platform (processing) on a computer terminal. The six subjects performed the nine locomotion modes (i.e., SLW, MLW, FLW, RD, SD, sit, stand, RA, and SA) in turn, as shown in Figure 3. Each locomotion mode lasted 120 s with a sampling frequency of 100 Hz, and each group of data involved six parameters, i.e., *X*-axis acceleration, *Y*-axis acceleration, *Z*-axis acceleration, *X*-axis angular acceleration, *Y*-axis angular acceleration, and *Z*-axis angular acceleration. During the experiment, owing to the wiring harness direction problem of Raspberry Pi, it needs to be mounted on the fixed structure connected with the lower limb using the binding to make sure the IMU is parallel to the lower limb. At this time, the *X*-axis is parallel to the lower limb and perpendicular to the ground. The *Z*-axis is parallel to the ground and same with the face direction. The *Y*-axis is perpendicular to the *X*-axis and *Z*-axis simultaneously.

Six subjects participated in this experiment to perform the nine locomotion modes in turn. The subjects were informed of the purpose and procedures of the study before signing the informed consent, and each subject gave consent for the use of their identifiable images. The experimental design was approved by the Ethics Committee of the Second Hospital of Jilin University, and the study was conducted in accordance with the Declaration of Helsinki.

### 3.2. Data Preprocessing

In this study, time windows of 100, 150, and 200 ms were used to sample the data, and the total data of six subjects were 50,000, 35,425, and 31,625, respectively. The data of six subjects were randomly divided into training and testing sets at a ratio of 8:2. The training sets needed to be reclassified before training the weight and threshold value of each IBPNN because the same locomotion mode had different labels in different IBPNNs. The testing sets did not participate in training the model to validate the recognition ability of the classification model facing completely new data.

**Figure 4:**
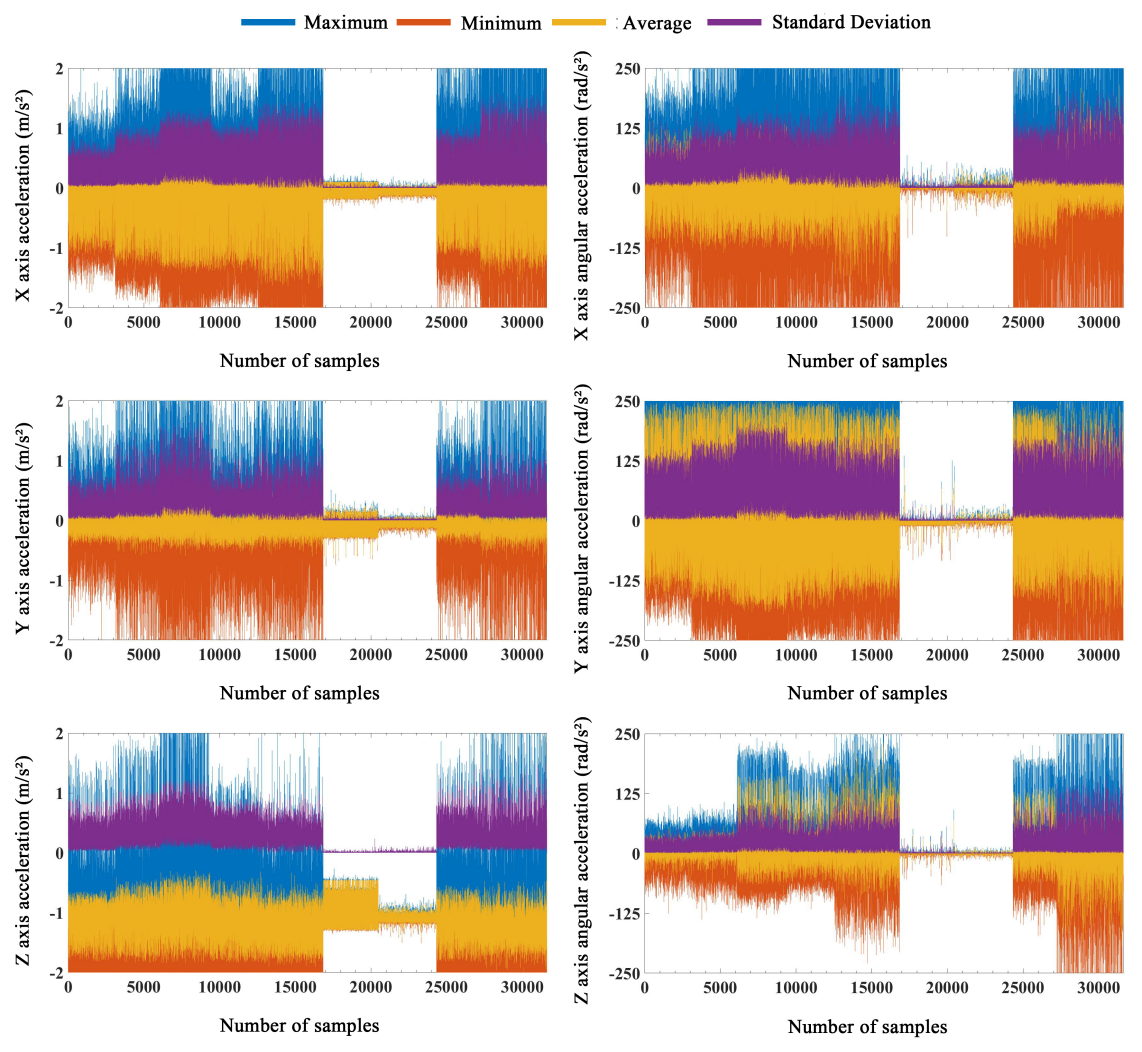
Time domain analysis for the 200-ms time window data (each locomotion mode included approximately 3500 samples arranged from left to right according the order in Figure 3).

For the data from the 200-ms time window, we performed time domain analysis (Figure 4) and selected the most representative and commonly used eigenvalues, including the maximum, minimum, average, and standard deviation. Note that the original values and eigenvalues after time domain analysis needed to be normalized before being input to the pattern recognition model.

### 3.3. Classification Model

We designed three classification models based on the waveform and time domain analysis of the original data obtained in the experiment to identify the nine locomotion modes, and the data were split at a ratio of 8:2 training and testing sets to train and validate the model. Here, the initial value of the IBPNN for each classification model was selected randomly, and the number of hidden layer neurons was set to 100. Each classification model is summarized as follows.

- IBPNN: Here, IBPNN is employed to identify all locomotion modes simultaneously. The input to the model is the original values and eigenvalues from the 200-ms time window, the number of IBPNN is only one, and the output is the nine locomotion modes. This type of model, which identifies all results once, has been applied in most locomotion mode recognition studies and has achieved good results [2,34,36].
- IBPNN-DTS A: Here, DTS A is employed with five IBPNNs as nodes to identify the nine locomotion modes hierarchically (Figure 1). The input to the model is the original values in the 200-ms time window, and the five IBPNNs can be trained simultaneously based on these original values. The first node divides the nine locomotion modes into four categories (a: SLW, MLW, FLW; b: RD, RA; c: SD, SA; d: sit, stand), and the other four nodes divide these four categories into the nine specific locomotion modes.
- IBPNN-DTS B: Here, DTS B is used with five IBPNNs as nodes to identify the nine locomotion modes hierarchically (Figure 2). The input to the model is the original values in the 100-ms, 150-ms, and 200-ms time windows, and the five IBPNNs can be trained simultaneously based on the original values, i.e., the same as DTS A. The first node divides the nine locomotion modes into two categories (a: sit, stand; b: others). The second node distinguishes the sit and stand modes, and the third node divides the class b into three categories (c: SD; d: SA; e: others). The fourth node divides class e into three categories (f: RA; g: RD; h: others), and the final node distinguishes SLW, MLW, and FLW from class h.

### 3.4. Structure and Parameter Optimization

We selected each IBPNN with 100 initial neurons in the hidden layer to improve the recognition effect of each classification model; however, too many neurons needed to optimize more number of the weight and threshold value, and the iteration time increased. Therefore, based on the 100 neurons, we performed experiments to reduce the number of neurons in the IBPNN and reduce the iteration time of the classification model without loss (or only small loss) of identification accuracy.

Generally, in the IBPNN algorithm, the initial weight and threshold value are generated randomly with uncertainty, and it is easy to make the classification model obtain a local optimal solution after several iterations at the initial position, which reduces identification performance. Here, we employed the ABC algorithm in this study to optimize the initial weight and threshold value of the IBPNN. The global search times were set to 10, the total number of bees was set to 200, and the times of local search was set to 1000 after the optimal value of each global search was identified. The combined global and local searches in the ABC algorithm allow the IBPNN to avoid falling into the local optimal situation in this iterative process, which improves the classification model’s identification performance.

## 4. Results and Discussion

### 4.1. Input to Classification Model

**Figure 5:**
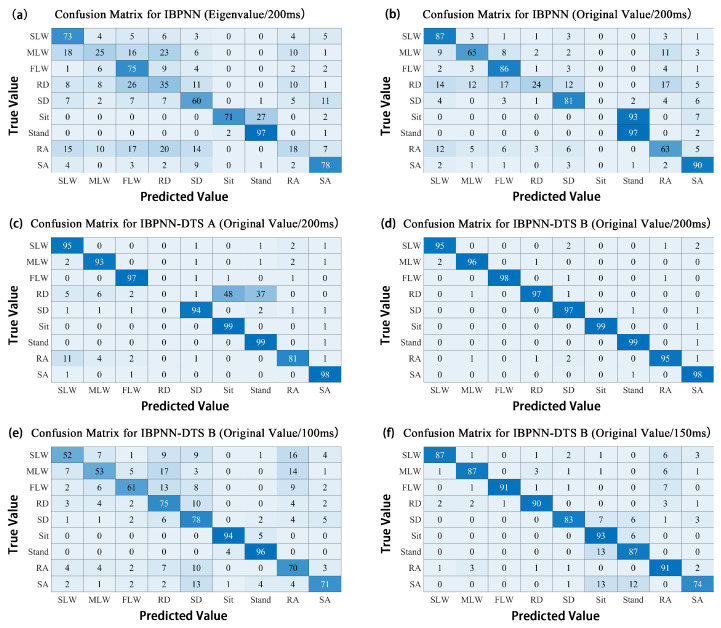
Confusion matrix for different inputs (all values are proportional).

**Figure 6:**
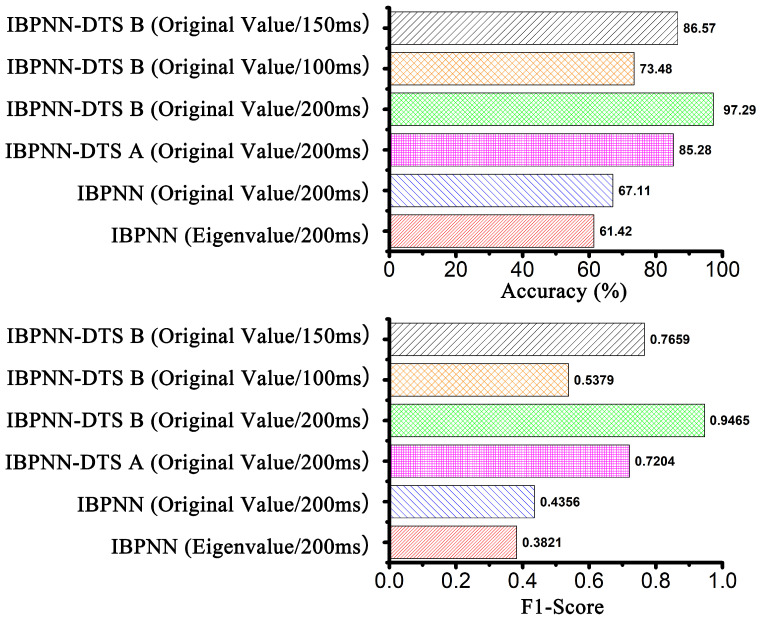
The evaluation index of different models with different input.

The eigenvalues of the collected data are frequently used as the input to a system model in most locomotion mode recognition studies. To the best of our knowledge, few studies have selected the original values. A single IMU with a short sampling time window was used in the current study; thus, it is impossible to assess the merits of the original values and subjective selection of eigenvalues. Therefore, we designed a comparative experiment to determine the best input to the model. First, the time window was 200 ms, and the IBPNN classification model used the original values and eigenvalues to identify the nine locomotion modes. Here, the selected eigenvalues (Figure 4) were the maximum, minimum, average, and standard deviation. After analyzing the confusion matrix in Figure 5, the accuracy and F1-Score (the higher value means the better robustness) of the IBPNN (Figure 6) based on the eigenvalues was 61.42% and 0.3821, while that based on the original values was 67.11% and 0.4356. From the confusion matrix shown in Figure 5a,b, we observe that the sit and stand modes could not be distinguished by the IBPNN based on the original values; however, overall accuracy was higher than the IBPNN based on eigenvalues.

**Figure 7:**
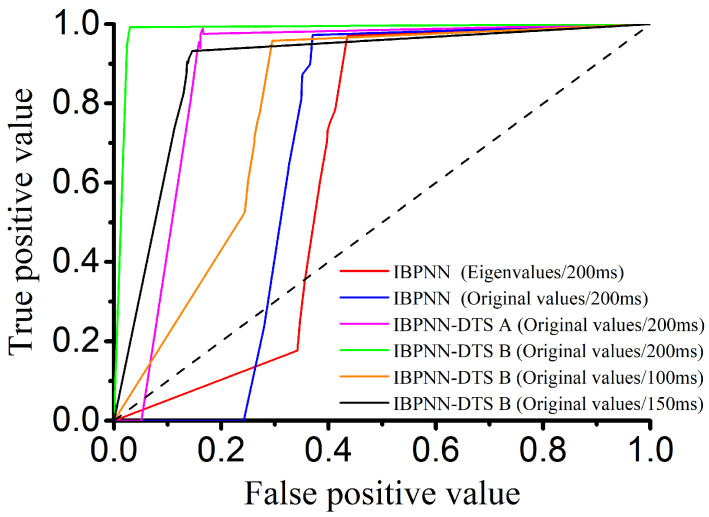
ROC curve of different models with different input (dashed line indicates completely random classification).

The IBPNN based on the original values with the simultaneous identification of the nine locomotion modes could not distinguish the sit and stand modes. There are two possible reasons for this phenomenon. The first reason is that the IBPNN classification model is unable to distinguish carefully between sitting and standing in the identification of nine locomotion modes. The other is that the original value of sit is much similar to that of stand with no feature. As a result, we classified these two motions based on both the eigenvalues and original values, and the accuracy and F1-Score of the IBPNN based on eigenvalues were 97.66% and 0.9536, while those based on the original values were 98.86% and 0.9774. These experimental results prove that the main reason for the inability to distinguish the sit and stand modes was that feature extraction for these modes was not sufficiently accurate enough when the IBPNN was used to identify all nine locomotion modes simultaneously. However, these two locomotion modes could be distinguished effectively based on the original values and had good robustness. As shown by the ROC curve in Figure 7, the IBPNN classification model demonstrates certain identification effects; however, some parts remain below the dashed line (completely random classification), which reflects failure of the IBPNN when identifying some data. Therefore, the original values were selected as the input to the classification model. In addition, the IBPNN classification model needed to be changed for better recognition ability.

### 4.2. Comparison of Classification Models

Unlike previous studies that only identified three to five locomotion modes [16,17], increasing the number of locomotion modes will make the features between different modes become less obvious. According to the analysis of the confusion matrix shown in Figure 5b, it was very easy to generate incorrect identifications between (SLW, MLW, FLW) and (RA, RD) and (SA, SD) and (sit, stand).

Therefore, based on using five IBPNNs as judgment nodes, we designed IBPNN-DTS A, as shown in Figure 1, to hierarchically identify the nine locomotion modes. The IBPNN-DTS A classification model effectively improved identification accuracy up to 85.28% and F1-Score up to 0.7204 (Figure 6). Although IBPNN-DTS A outperformed the IBPNN, the IBPNN-DTS A classification model was not sufficiently precise compared with existing methods [6,9], which have demonstrated accuracy of greater than 93%, and there is still failure of the part which is under the dashed line according to the ROC curve shown in Figure 7. As a result, the IBPNN-DTS A classification model did not completely satisfy the requirements of locomotion mode recognition.

The confusion matrix for the IBPNN-DTS A is shown in Figure 5c, and the results indicate that miscalculation between level walking, RA, RD, sit, and stand persists. Thus, without increasing the number of nodes, we designed the IBPNN-DTS B classification model (Figure 2). The experimental results shown in Figure 6 demonstrate that the IBPNN-DTS B classification model obtained overall accuracy of 97.29% and high F1-Score of 0.9465, which is more precise than both the IBPNN-DTS A and IBPNN models. The IBPNN-DTS B confusion matrix shows Figure 5d illustrates that this model can effectively and accurately identify all nine locomotion modes and has high robustness.

### 4.3. Time Window Selection

According to the selection of the time window problem, we conducted simple preliminary experiments using the 200, 400, 600, 800, 1000, and 1200-ms time windows that are less than a gait cycle. The experiments show that when a time window is less than 1000 ms, with the longer time the window, the accuracy of the classification model showed an ascendant trend, and when the time window is more than 1000 ms, the accuracy of the classification model began to decline. The results indicate that lengthening the time window is helpful in improving accuracy. The time window of the locomotion mode recognition system also depends on the requirements of the control system, which is generally less than 200 ms for better system performance. In this study, an initial time window of 200 ms was selected, which is less than the 250-ms time window employed in a previously reported backpropagation neural network [31]. Here, to verify the identification effect of different time windows on the classification model, the length of the time window was reduced to 150 and 100 ms under the condition that the original values were taken as input and the classification model adopted the IBPNN-DTS B with the highest accuracy. The ROC curve shown in Figure 7 indicates that the curve of IBPNN-DTS B under different time windows is entirely above the dashed line. As shown in Figure 6, the accuracy and F1-Score of IBPNN-DTS B with a 150-ms time window were 86.57% and 0.7659, while those with a time window of 100 ms were only 73.48% and 0.5379. The experimental results shown in Figure 5e,f indicate that the data in the 150-ms time window resulted in misjudgment of sit, stand, and RA, while that in the 100-ms time window had a large number of misjudgments in seven locomotion modes with the exception of the sit and stand modes.

Since the accuracy of locomotion mode recognition algorithms is closely related to human safety, it is acceptable to extend the time window to ensure human safety. Therefore, for a single IMU, the data in the 200-ms time windows are considered the most effective and safe for human movement relative to the assessment of locomotion modes.

### 4.4. Structure and Parameter Optimization Results

**Figure 8:**
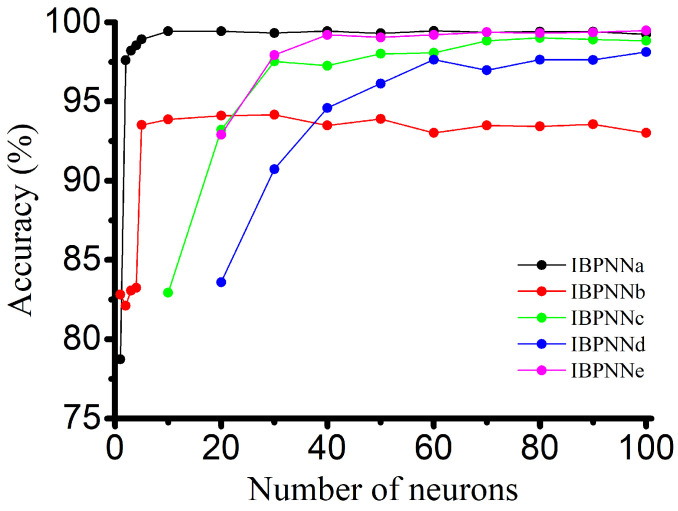
Neuron number optimization.

A large number of neurons, which means higher weights and threshold value, have significant impact on the system relative to storage, transfer, or iteration of these parameters. In this study, the number of initial hidden layer neurons was set to 100 to ensure sufficient recognition ability for each classification. While maintaining sufficient precision, the number of neurons was reduced to reduce the number of the weight and threshold value in order to improve the performance of the overall classification model system. The experimental results shown in Figure 8 demonstrate that the accuracy of each IBPNN has an inflection point as the number of neurons is reduced. If the number of neurons is less than this inflection point, accuracy will be reduced rapidly, and the model will lose its recognition ability. The optimized results of the number of hidden layer neurons for the five IBPNN with different functions are listed in Table 2.

**Figure 9:**
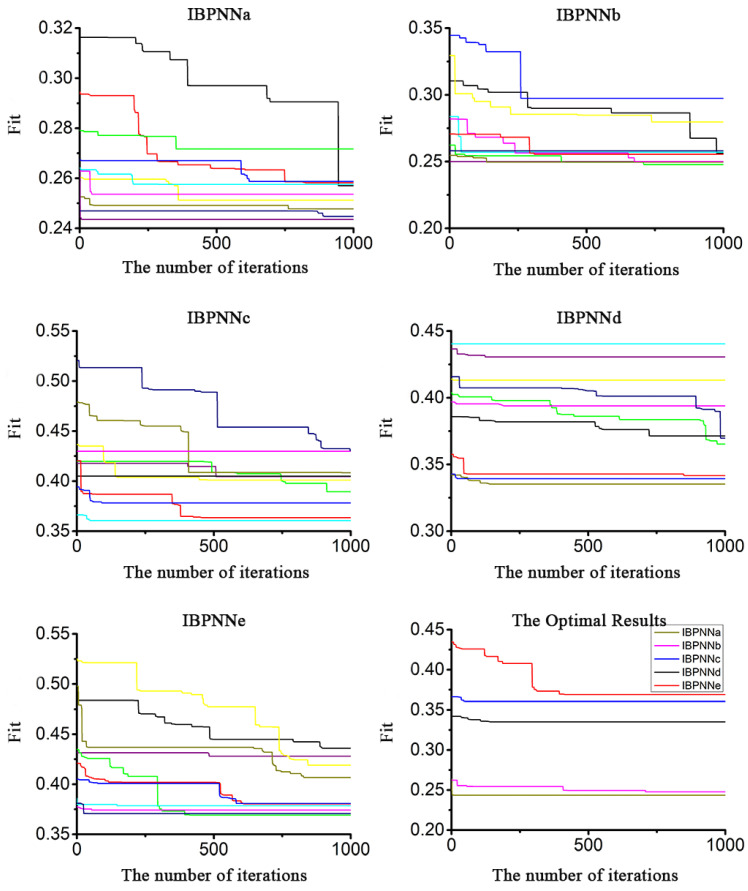
Initial weight and threshold value optimization with artificial bee colony (ABC) (each color represents a single global search).

During the experimental process, we found that some randomly generated initial weight and threshold values made the system not converge after only a few iterations, which increased system uncertainty. To address this problem, we employed the ABC algorithm to optimize the initial weight and threshold value of five optimized IBPNNs. During this optimization process for the IBPNNa to IBPNNe shown in Figure 9, the weight and threshold value of the five IBPNNs were globally searched 10 times (refer to the different colored curves) and locally searched 1000 times after determining the optimal value in each global search to avoid falling into a local optimal situation. The optimization result curve is shown in the last picture in Figure 9.

**Figure 10:**
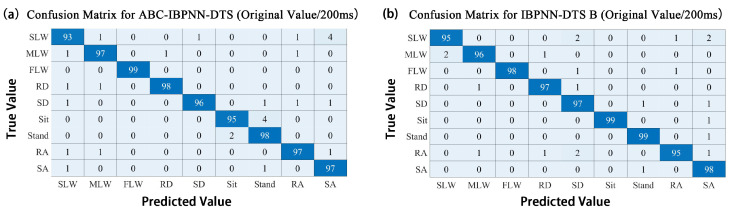
Confusion matrix for IBPNN-DTS B models before and after optimization (all values are proportional).

**Figure 11:**
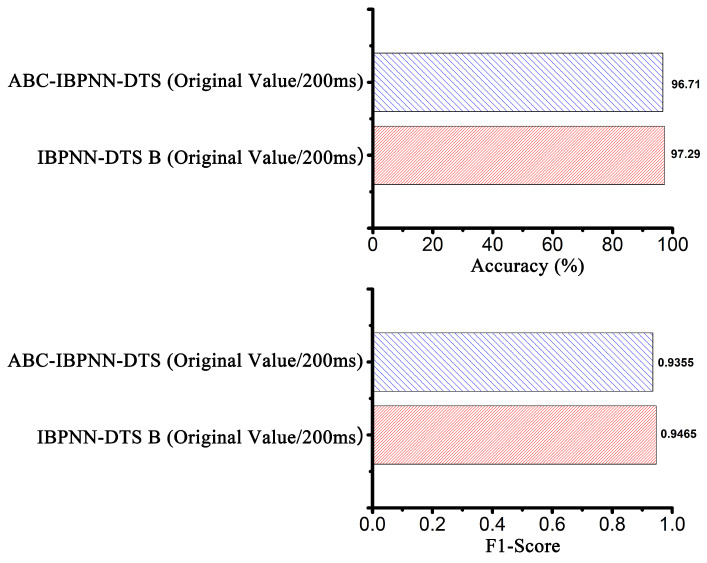
The evaluation index of IBPNN-DTS B models before and after optimization.

**Figure 12:**
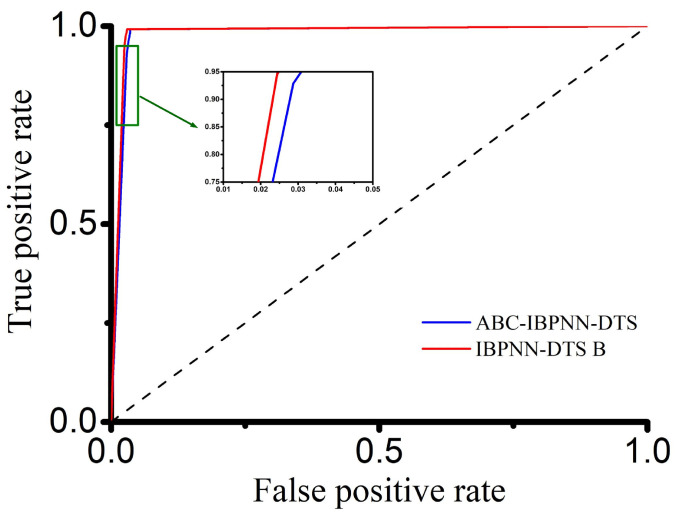
ROC curve of IBPNN-DTS B models before and after optimization.

The confusion matrix for the ABC-IBPNN-DTS classification model obtained with a reduced number of neurons in the hidden layer, initial weight, and threshold value optimization is shown in Figure 10. As can be seen, accuracy reached 96.71% and F1-Score achieved the value of 0.9355. Compared to the IBPNN-DTS B without optimization, the number of parameters in the classification model shrank by 66% based on structure optimization; however, accuracy was reduced by only 0.58% keeping high robustness (Figure 11). Meanwhile, from the ROC curve shown in Figure 12, the curve of ABC-IBPNN-DTS is above than the dashed line.

In addition, we simulated the running state of the classification models in practical application by using Visual C++ 6.0 on a computer (Intel Core i5-9400F CPU 2.90 GHZ). The running speed of IBPNN-DTS A, IBPNN-DTS B, and ABC-IBPNN-DTS are 423 μs, 986 μs, and 437 μs, respectively. The simulation results show that ABC-IBPNN-DTS can satisfy the real-time requirements of the system with the similar running speed of IBPNN-DTS A.

## 5. Conclusions

In this paper, to reduce the burden of sensors on users and realize the identification of more locomotion modes at high accuracy, we analyzed the locomotion mode experimental data and proposed the ABC-IBPNN-DTS classification model based on the original values of a single IMU to hierarchically identify nine common locomotion modes, i.e., SLW, MLW, FLW, RD, SD, sit, stand, RA, and SA. Table 3 shows the comparison between our method and state-of-the-art methods in the field of locomotion mode recognition. We only used a single IMU rather than multiple sensors, as conventionally used [6,7,8]. This strategy can avoid the increased computation cost due to the fusion of mixed signals and reduce extra burden on a user’s body. Since feature extraction can seriously affect the accuracy of the model [28], our method can automatically extract the features in the data, which can reduce subjective error. In addition, on the basis of achieving high-accuracy, the DTS we designed can hierarchically identify up to nine locomotion modes more than the methods with one-time recognition [6,7,8,17,28].

In an experiment, we compared the influence of the original values and eigenvalues of IMU on identification accuracy. We found that the effect of using the original values is better than that based on subjective selection of the eigenvalues. After determining the original values as the model input, we compared the identification ability of different classification models (IBPNN, IBPNN-DTS A, and IBPNN-DTS B), and the experimental results demonstrated that the recognition accuracy of IBPNN-DTS B was the best. To ensure good results under different time windows, time windows of 200, 150, and 100 ms were investigated in a comparative experiment, and the results demonstrated that a shorter time window with the fewer features makes more miscalculation in locomotion mode recognition. We found that the IBPNN-DTS classification model with IBPNN-DTS B based on the original values with a time window of 200 ms demonstrated the best recognition accuracy (97.29%).

By adopting the ABC-IBPNN-DTS classification model with structure optimization for reducing the number of parameters in IBPNN and initial weight and threshold value optimization by ABC algorithm for eliminating system uncertainty and avoiding randomly generated initial value tend to result in a failure to converge or falling into local optima, it achieved better performance when detection accuracy and F1-Score stay the same level. The experimental results have demonstrated that the recognition accuracy of ABC-IBPNN-DTS can reach up to 96.71%, and compared to IBPNN-DTS without optimization, the number of parameters in ABC-IBPNN-DTS shrank by 66% with only a 0.58% reduction in accuracy while the classification model kept high robustness. Thus, with the ABC-IBPNN-DTS classification model based on a single IMU with low burden located under the knee joint, wearable robotic system, especially active lower limb prostheses, can rapidly and effectively realize man–machine interaction, which is expected to bring convenience to people. In our future work, we will focus on the implementation of on-board training for real-time locomotion mode recognition to adapt to complex actual conditions.

## Figures and Tables

**Table 1:** Velocity and experimental platform of locomotion modes.

Locomotion Modes	Velocity	Platform
SLW	3 km/h	Treadmill
MLW	4.2 km/h	Treadmill
FLW	6 km/h	Treadmill
RD	4.2 km/h	9 degrees ramp on treadmill
SD	Slow	Stairs
Sit	Slow	Chair
Stand	Slow	Chair
RA	4.2 km/h	9 degrees ramp on treadmill
SA	Slow	Stairs

**Table 2:** Function and number of neurons for five improved backpropagation neural networks (IBPNNs).

IBPNN	Function	Number of Neurons
IBPNNa	Distinguish between (sit, stand)and (others)	10
IBPNNb	Distinguish between sit and stand	30
IBPNNc	Distinguish between SD, SA and (the others)	30
IBPNNd	Distinguish between RD, RA and (the others)	60
IBPNNe	Distinguish between SLW, MLW, FLW	40

**Table 3:** Comparison with state-of-the-art methods.

Reference	Sensors	Feature Extraction	Classifier	Number of Locomotion Modes	Accurary
Young et al. 2014 [6]	3 IMUs and 1 pressure sensor	Manual	LDA	5	93.9%
Young et al. 2014 [7]	1 IMU and 1 axial load cell.etc	Manual	Dynamic Bayesian Network	5	94.7%
Liu et al. 2017 [8]	1 IMU and 2 pressure sensors	Manual	HMM	5	95.8%
Feng et al. 2019 [17]	1 strain gauge	Automatic	CNN	3/5	92.53%/89.11%
Gao et al. 2020 [28]	1 IMU	Manual	Terrain Geometry	5	98.5%
Our Method	1 IMU	Automatic	IBPNN-DTS B/ ABC-IBPNN-DTS	9	97.29%/ 96.71%

## Data Availability

The data presented in this study are available on request from the corresponding author.
